# Three-dimensional model of glioblastoma by co-culturing tumor stem cells with human brain organoids

**DOI:** 10.1242/bio.056416

**Published:** 2021-02-22

**Authors:** Roberta Azzarelli, Michela Ori, Anna Philpott, Benjamin D. Simons

**Affiliations:** 1 Wellcome - MRC Cambridge Stem Cell Institute, Jeffrey Cheah Biomedical Centre, Cambridge Biomedical Campus, University of Cambridge, Cambridge, CB2 0AW, UK; 2 Department of Oncology, University of Cambridge, Hutchison/MRC Research Centre, Cambridge Biomedical Campus, Cambridge CB2 0XZ, UK; 3 Department of Biology, Unit of Cell and Developmental Biology, University of Pisa, S.S. 12 Abetone e Brennero 4, 56127 Pisa, Italy; 4 The Wellcome Trust - Cancer Research UK Gurdon Institute, University of Cambridge, Tennis Court Road, Cambridge CB2 1QN, UK; 5 Department of Applied Mathematics and Theoretical Physics, Centre for Mathematical Sciences, University of Cambridge, Wilberforce Road, Cambridge, CB3 0WA, UK

**Keywords:** Neural stem cells, Glioblastoma, Organoid, Neurogenesis, 3D culture, Brain tumor

## Abstract

Emerging three-dimensional (3D) cultures of glioblastoma are becoming powerful models to study glioblastoma stem cell behavior and the impact of cell–cell and cell–microenvironment interactions on tumor growth and invasion. Here we describe a method for culturing human glioblastoma stem cells (GSCs) in 3D by co-culturing them with pluripotent stem cell-derived brain organoids. This requires multiple coordinated steps, including the generation of cerebral organoids, and the growth and fluorescence tagging of GSCs. We highlight how to recognize optimal organoid generation and how to efficiently mark GSCs, before describing optimized co-culture conditions. We show that GSCs can efficiently integrate into brain organoids and maintain a significant degree of cell fate heterogeneity, paving the way for the analysis of GSC fate behavior and lineage progression. These results establish the 3D culture system as a viable and versatile GBM model for investigating tumor cell biology and GSC heterogeneity.

This article has an associated First Person interview with the first author of the paper.

## Abstract (precis)

**Summary:** We describe a method to co-culture glioblastoma stem cells with human cerebral organoids in order to model tumor complexity and recapitulate cellular heterogeneity *in vitro*.

## INTRODUCTION

Glioblastoma multiforme (GBM) is the most common and aggressive form of primary brain tumor. The majority of GBMs form rapidly from normal brain cells, while around 10% are secondary to low-grade astrocytomas (Louis et al., 2016). Current therapies include surgical removal of the bulk of the tumor, combined with radiation therapy and chemotherapy aimed at killing the remaining cells that have infiltrated the parenchyma. However, despite treatment, GBM usually recurs, contributing to extremely poor patient survival (3–7%) beyond 5 years (Aldape et al., 2019). To advance our understanding of tumor biology and develop new therapeutic strategies, it is essential to develop *in vitro* models that mimic as much as possible the *in vivo* tumor (Aldape et al., 2019).

To date, a typical feature that is difficult to recapitulate *in vitro* is the heterogeneity in cell types that characterizes GBMs in their native environment (Robertson et al., 2019). Immunochemical and molecular analysis of GBM specimens, including more recent data from single-cell transcriptomic analysis (Couturier et al., 2020; Neftel et al., 2019; Patel et al., 2014; Tirosh et al., 2016) revealed the presence of distinct differentiated neural and glial tumor cell types as well as their immature proliferating precursors. This snapshot of cell type heterogeneity is likely to reflect the unfolding of a dynamic process of lineage progression, which would then result in the apparent simultaneous presence of distinct developmentally-related and temporally asynchronous cells along the lineage (Azzarelli et al., 2018b; Couturier et al., 2020; Lan et al., 2017; Lu et al., 2019; Swartling et al., 2015). The identification and isolation of a small subpopulation of stem-like cells in brain tumors (Galli et al., 2004; Pollard et al., 2009; Singh et al., 2004) supports this idea, as does more recent work that traced the behavior of barcoded glioblastoma cells upon serial xenotransplantation (Lan et al., 2017). This study provided evidence that GBM is supported by a proliferative hierarchy, reminiscent of a normal developmental program, in which a subpopulation of stem-like cells give rise to progenitors with more limited proliferative potential. These findings suggest that stem-like cells may function as tumor-initiating cells during relapse, identifying them as potential targets for therapy. At the same time, understanding the potential link between tumor cell fate and normal developmental dynamics may identify new therapeutic strategies that target differentiation rather than proliferative programs.

**Fig. 1.**
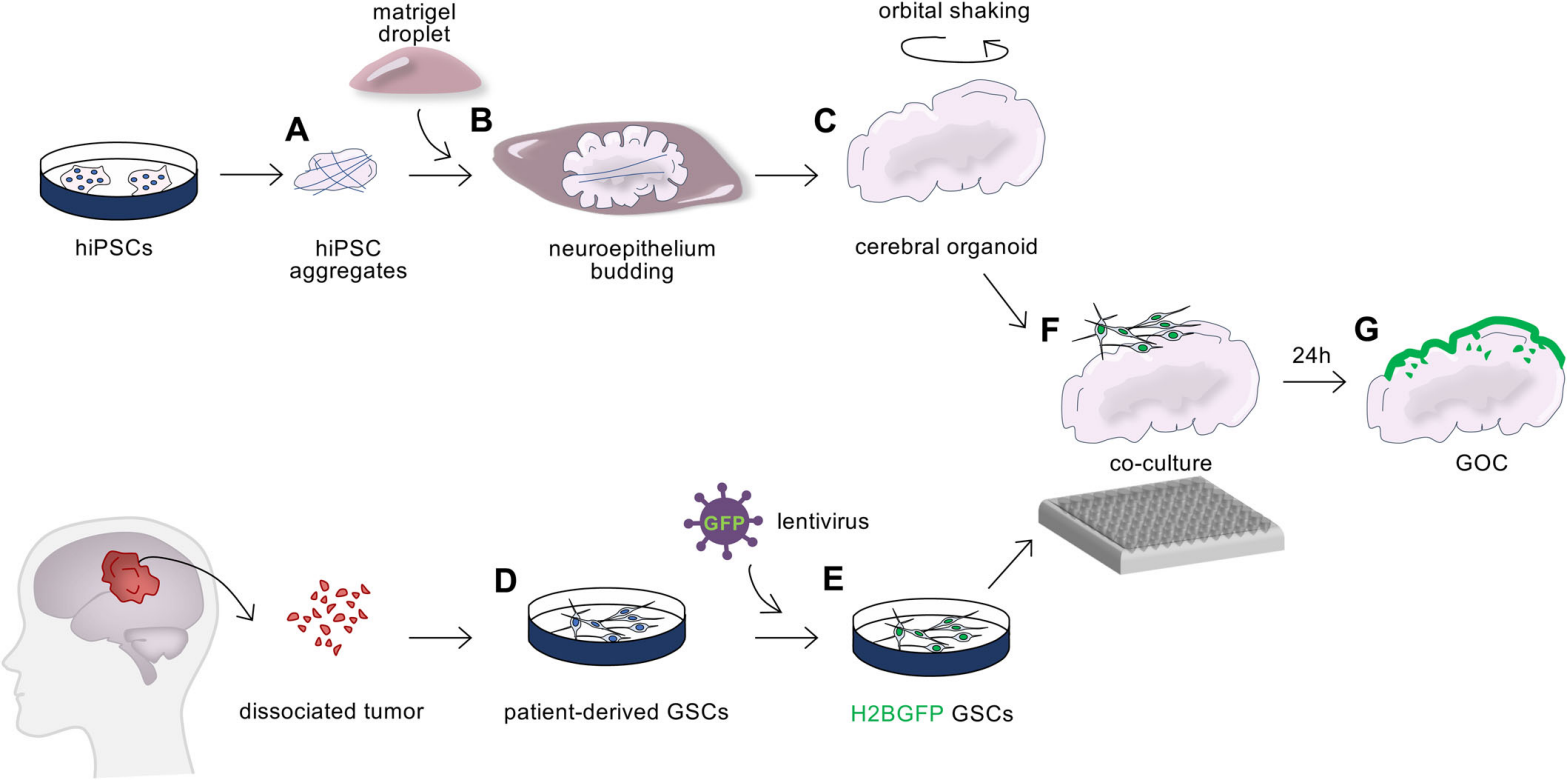
**Workflow of the co-culture method.** Schematic outline of the different steps of the protocol. (A) Human induced pluripotent stem cells (hiPSCs) are seeded on microfibers in neural induction media to form embryoid body-like aggregates. (B) Aggregates are then embedded in Matrigel droplets, which favor inversion of the polarity and protrusion of the neuroepithelium. (C) Subsequent removal of the Matrigel and culture in maturation media on an orbital shaker result in expansion and maturation of the organoids. (D) In parallel, patient-derived GSCs are expanded in 2D adherent cultures and (E) infected with lentiviruses to express the fluorescent histone marker H2B-GFP. (F) H2B-GFP GSCs are then co-cultured with human brain organoids in a low adherence 96-well plate for 24 h, generating glioblastoma-organoid co-cultures (GOC) (G).

Glioblastoma stem cells (GSCs) have been isolated and grown in two-dimensional (2D) monolayer cultures by several laboratories (Galli et al., 2004; Lee et al., 2006; Pollard et al., 2009; Singh et al., 2004). The use of these lines to study the biology of GBM entails several practical advantages: this includes the availability of established cell lines to researchers that do not have direct access to patient biopsies; and the fact that, in culture, cell populations are generally homogenous, and accessible to bulk cellular and molecular analysis. However, such 2D cultures do not fully recapitulate the complexity of the *in vivo* tumor. Emerging research in the field has shown that growing fragments of glioblastoma biopsies or GSCs in three-dimensional (3D) cultures can maintain a certain degree of cell heterogeneity (Hubert et al., 2016; Ogawa et al., 2018; Pine et al., 2020); can preserve the genetic alterations of the original tumor better than 2D cultures (Jacob et al., 2020; Linkous et al., 2019); and can recapitulate some cell–cell and cell–microenvironment interactions found *in vivo* (Hubert et al., 2016; Krieger et al., 2020; Pine et al., 2020; Zhu et al., 2020; for reviews see Azzarelli, 2020; Gomez et al., 2019; Silvia and Dai, 2020). However, it is not clear whether the conditions of previous *in vitro* culture models can sustain the simultaneous presence of progenitors and their differentiating progeny to better mimic the *in vivo* situation. Moreover, some culture models do not allow investigation of the interaction between cancer and non-cancer cells, nor invasion of the normal surrounding tissue by the cancer cells. Here, to overcome these limitations, we describe a method to model GBM in 3D by co-culturing GSCs with cerebral organoids and study cell fate identity as tumor cells invade the organoids. This protocol involves multiple coordinated steps, including the generation of 3D human cerebral organoids, and the parallel growth and fluorescent labelling of patient-derived GSCs, followed by co-culture of the two (Fig. 1).

Here we characterize the fate identity of GSCs once they are integrated into the organoids to show that our model can sustain the simultaneous presence of stem/progenitor cells and their differentiated progeny. This is in line with a recent report showing that amongst all the different GBM models, co-culture with brain organoid maintains GSCs in a neural progenitor-like cell state that is associated with stem cell activity and recapitulates the cellular plasticity of the original tumors (Pine et al., 2020).

Together with previous works (Goranci-Buzhala et al., 2020; Krieger et al., 2020; Linkous et al., 2019; Pine et al., 2020), our *in situ* analysis of the cellular differentiation state of GSCs indicates that our model is suitable for studies of cancer stem cell heterogeneity and lineage progression, as well as the investigation of the relation between cancer cells and the normal surrounding tissue. By recapitulating the complexity of the *in vivo* tumor, this model can provide a versatile platform to test novel therapies, and will thus help bridge the gap between the bench and the clinic.

## RESULTS

### Generation of cerebral organoids from different human induced pluripotent stem cell lines

**Fig. 2.**
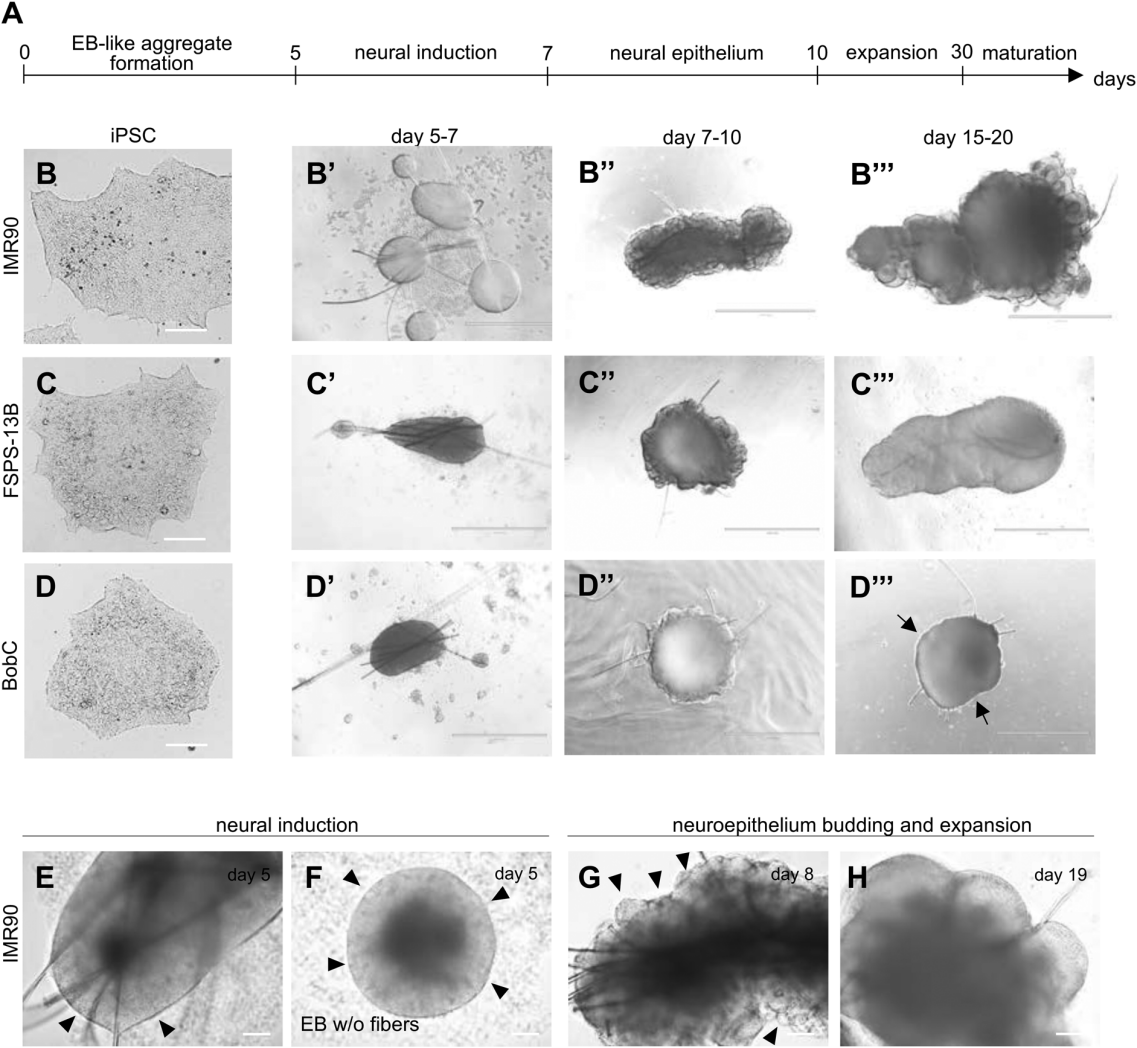
**Generation of cerebral organoids from different induced pluripotent stem cell (iPSC) lines.** (A) Timeline of the differentiation process. (B–D) Induced pluripotent stem cell lines IMR-90 (B), FSPS-13B (C) and BobC (D) have been differentiated to generate cerebral organoids. Representative images are shown for the different stages of the protocol from at least three different experiments: B′,C′,D′, neural induction on microfibers; B″,C″,D″, neuroepithelial budding upon Matrigel encapsulation; B‴,C‴,D‴, organoid expansion and maturation (black arrows in D‴ indicate the smooth edges of BobC-derived organoids, which are failing to develop). (E–H) Differentiation of IMR-90 cells with (E) or without (F) microfibres showing neural induction (E,F, black arrowheads show the bright appearance of the neuroepithelium) and correct formation and expansion of the neuroepithelial bulges (G,H, black arrowheads in G indicate budding epithelial structures). EB: embryoid body. Scale bars: B–D 100 µm; B′–B‴,C′–C‴,D′–D‴ 1000 µm; E–H 100 µm.

To generate cerebral organoids, we followed the published protocol from Lancaster and Knoblich, referring to (Lancaster et al., 2017) for details (Fig. 2A). Here we use three different human induced pluripotent stem cell (iPSC) lines, BobC, FSPS-13B and IMR-90; all of them grew in separated colonies characterized by flat undifferentiated morphology (Fig. 2B,C,D). We dissociated iPSCs and seeded 9000 cells/well in a low adherence 96-well plate together with microfibers, as described previously (Lancaster et al., 2017). After 5–7 days, cells aggregated along the microfibers and underwent a process of neural induction, resulting in the formation of an outer epithelium, the neuroectoderm (Fig. 2B′,C′,D′). After neural induction, cells were embedded in Matrigel, which induced an inversion of the polarity and the formation of the neuroepithelium. Neuroepithelial bulges started to emerge from the organoid surface at around 7–8 days in culture and expanded over time (Fig. 2B″,C″,D″). After day 10, organoids were then grown in a media that promotes cell differentiation, organoid expansion and maturation (Fig. 2B‴,C‴,D‴).

We found that the cell line IMR-90 generated high-quality organoids, characterized by the bright appearance of the neuroectodermal layer (Fig. 2E,F; Fig. S1A,B, black arrowheads), efficient budding of several neuroepithelial structures (Fig. 2G; Fig. S1C, black arrowheads), and their expansion over time (Fig. 2H; Fig. S1D). The line FSPS-13B generated some organoids showing robust differentiation (Fig. 2C‴), while others failed to develop cortical-like structures (Fig. S1E–H). Our observations indicate that the organoids that did not develop were failing to differentiate properly into neuroectoderm and neuroepithelium at earlier stages of the protocol. Indeed, during neural induction and neuroectoderm formation, the outer epithelial layer of these cultures looked darker and displayed irregular bulges (Fig. S1E, black arrowheads), instead of showing the classic brighter appearance. This was perhaps more evident when looking at neural induction in embryoid bodies without microfibers (Fig. 2F; Fig. S1B,SF). Such irregularities on the organoid surface are often associated in the next stages with delamination of cells that could be of neural crest origin (Fig. S1G,H). Indeed correct neuroepithelial budding as shown in Fig. 2G and H was impaired by the presence of these non-neuronal/neural crest-like cells that will eventually outgrow the neuroepithelial stem cells and cover the entire organoid surface (Fig. S1H).

While the line FSPS-13B was still capable of generating well-organized cerebral organoids, the BobC line formed only very small neuroepithelial budding structures, which degenerated and resulted in organoids that were smaller and with smooth edges (Fig. 2D‴, black arrows indicate the smooth organoid surface). Thus, since some iPSC lines are more capable of generating brain organoids than others, it is essential to consider specific morphological parameters to assess organoid quality before co-culturing tumor cells.

### Lineage analysis of cerebral organoids shows neural progenitors and neuronal differentiation

The quality of the cerebral organoids was also assessed by immunochemistry (results are from lines IMR-90 and FSPS-13B). At day 14, we found that cells self-organize in neural rosette-like structures, which are positive for the adherence junction epithelial marker N-cadherin (Fig. S2A,B, white arrows indicate rosette structures). Accumulation of N-cadherin towards the presumptive lumen of the rosette indicates the establishment of a polarity similar to that found in the neural tube *in vivo*. Cells in these structures were negative for the endodermal marker SOX17 (Fig. S2A,B), and positive for the neural progenitor markers SOX2 and PAX6 (Fig. S2C,D), the latter of which also marks dorsal forebrain identity at early neurodevelopmental stages. Thus, our organoids displayed appropriate forebrain neuroepithelial identity. We also looked at the expression of these markers in ‘suboptimal’ organoids to examine their spatial organization and understand better the reason behind organoid failure. We found the presence of SOX2+/PAX6+ progenitors in suboptimal organoids, indicating the correct acquisition of forebrain neural identity (Fig. S2E–G). However, these cells were not always organized in rosettes (Fig. S2E′–G′, white arrowheads indicate rosettes and white arrows indicate non-structured regions), and the SOX2+/PAX6+ areas were surrounded by a non-neural region, which may inhibit further development into cerebral cortex-like structures.

Organoids that exhibited correct neuroepithelial budding were kept in organoid maturation media for long periods of time, and produced prominent cortical-like structures from day 30–32 (Fig. S2H–K). At this stage, our cerebral organoids expressed both neural progenitor and differentiated neuron markers with clear spatial segregation of the two cell populations (Fig. S2H–K). SOX2+ and PAX6+ progenitors were found closer to a ventricular-like zone (Fig. S2H–K, white arrows), while TUJ+ neurons had migrated towards the outer organoid surface (Fig. S2H,I, white arrowheads), recapitulating *in vivo* cortical plate formation. Moreover, these TUJ+ neurons also expressed markers typical of early born deep layer cortical neurons such as TBR1 (Fig. S2J,K, white arrowheads), confirming correct cortical identity.

### Optimization of glioma stem cell integration into brain organoids

**Fig. 3.**
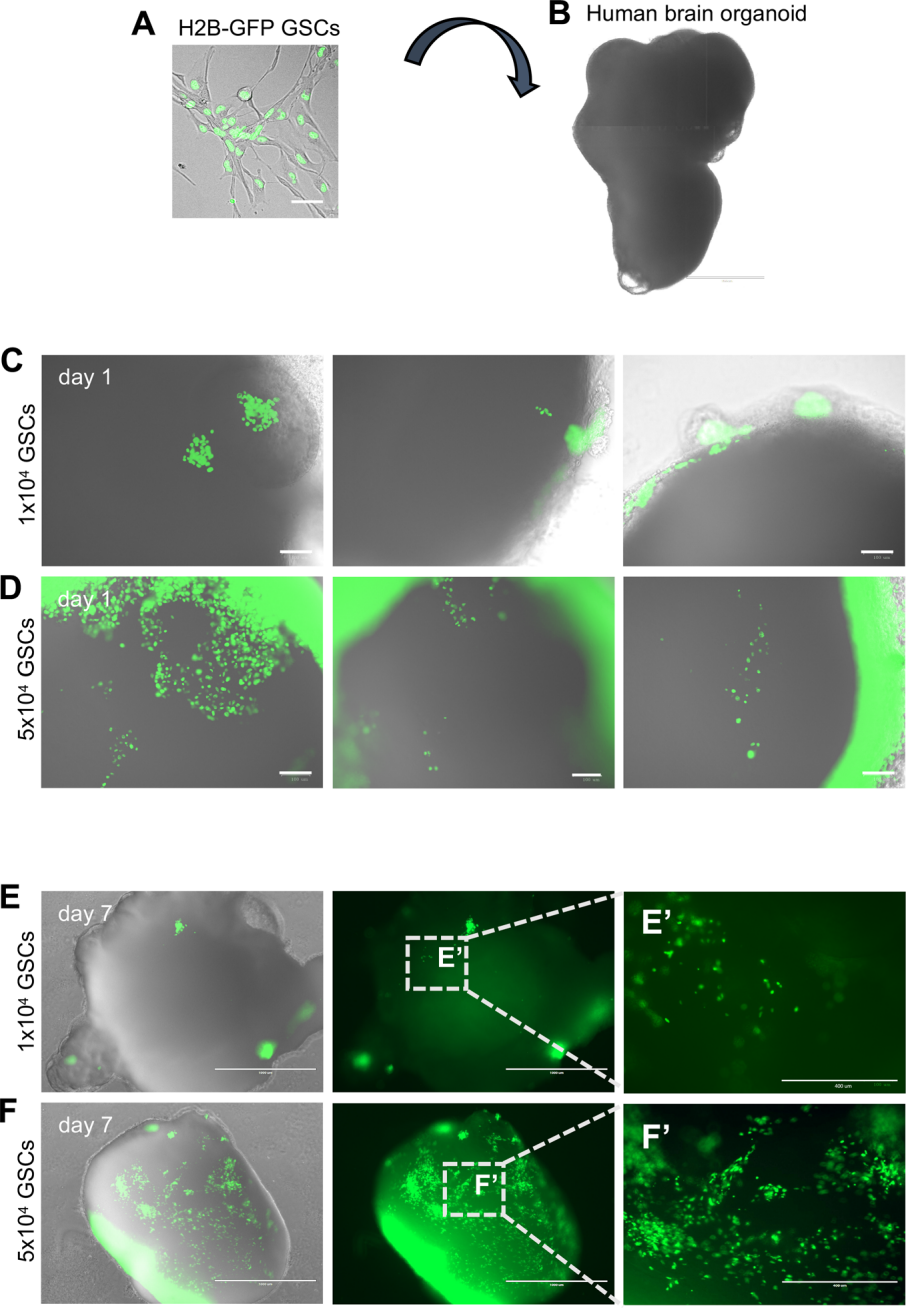
**Co-culture of** **GSCs** **(line GCGR-E27) and brain organoids.** (A,B) Experimental outline, showing GSCs infected with lentiviruses carrying H2B-GFP (A) and a 42-day-old human brain organoid (B). (C–F) Representative images of H2B-GFP GSCs (in green) invading brain organoids (in grey) at 1 day (C,D) and 7 days after co-culture (E,F). The number of GSCs used for the co-culture is indicated. *n*≥3 organoids. Scale bars: A 50 µm; B 1000 µm; C,D 100 µm; E,F 1000 µm; E′,F′ 400 µm.

Patient-derived GSCs can be expanded in 2D in serum free conditions supplemented with EGF and FGF2 (Conti et al., 2005; Pollard et al., 2009, 2006). In order to visualize GSCs upon integration into organoids, we explored ways to mark cells with a fluorescent marker (Fig. 3A; Fig. S3). We tried different transfection approaches using Lipofectamine and the less toxic reagent TransIT-LT1, but only a limited number of cells were marked and the overall cell survival was low (Fig. S3A,B). We thus opted for lentiviral infection, which resulted in approximately 80% of the cells expressing the histone marker H2B-GFP (Fig. S3C). We also noted that cell survival was higher if cells were infected without using the polybrene reagent, which is often added to enhance virus uptake (Fig. S3C,D). We then co-cultured different densities of H2B-GFP+ GSCs with 42-day-old mature brain organoids (Fig. 3B), using a small format in 96-well plates that should favor cell integration. We used two different patient-derived cell lines, GCGR-E27 (Fig. 3) and GCGR-E35 (Fig. S4), both classified as RTKI, the closest classification to pro-neural (GSC lines were obtained from the Glioma Cellular Genetic Resource, University of Edinburgh, gcgr.org.uk). We transferred organoids into a well of low adherence 96-well plates (one organoid per well) and added either 1×10^4^ or 5×10^4^ GSCs marked with H2B-GFP. After overnight incubation, the organoids were transferred back to a 6 cm dish, thus removing the GSCs that did not integrate. At one day of co-culture, we observed GFP+ cells on the surface of the organoids in different areas, showing efficient GSC engraftment at both cell concentrations (Fig. 3C,D; Fig. S4A,B). We then collected organoids at 7 days after initial co-culture and found that GSCs had colonized the organoids forming a glioblastoma-organoid co-culture (GOC) (Fig. 3E,F; Fig. S4C,D). When co-culturing with 1×10^4^ GSCs, we could see some individual cells dispersed sparsely across the organoids (Fig. 3E,E′), a feature that could be useful for subsequent analysis of clonal behavior, if combined with real time live-imaging to follow single cells. In contrast, organoid invasion by interconnected streams of cells (Fig. 3F,F′) was more prominent with co-culture of 5×10^4^ GSCs, and we would recommend to use this cell density when studying GSC cell migration and invasion. Interestingly, the behavior of the two cell lines was highly comparable (Fig. 3; Fig. S4), probably reflecting their relatively similar genetic profile, since both lines are classified as RTKI and are characterized by no PDGFRα amplification (Dr Gillian Morrison and Professor Stephen Pollard, personal communication).

### Effect of the brain organoid environment on glioma stem cell differentiation

One of the advantages of modelling GBM in 3D using GOC is to expose cancer stem-like cells to an *in vivo*-like environment that could result in the recapitulation of the GBM cellular heterogeneity. Therefore, we investigated the fate of GSCs to see whether our model could indeed support simultaneous presence of different GSC-derived cell types.

**Fig. 4.**
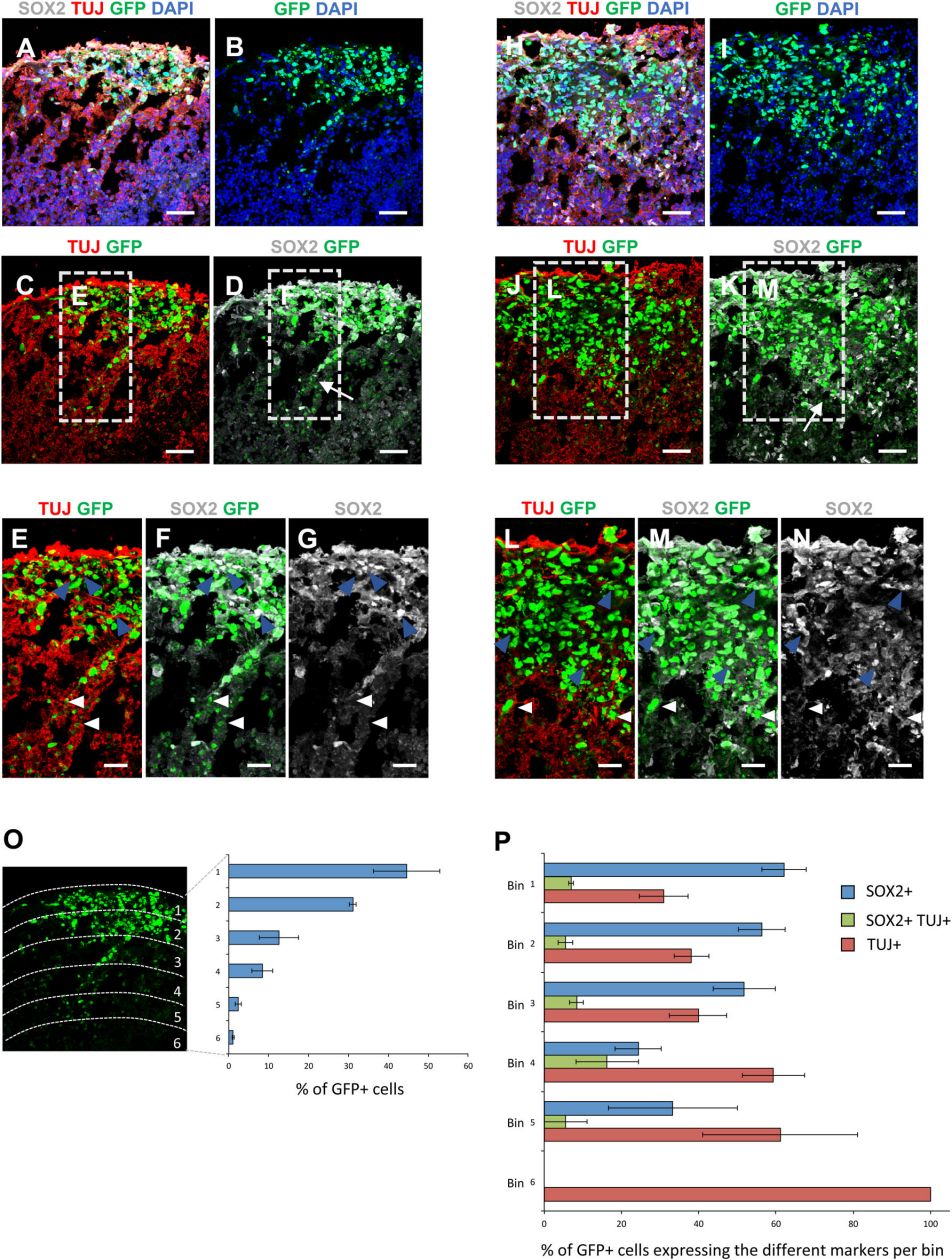
**Fate identity of** **GSCs** **in organoids.** (A–N) Immunostaining to detect TUJ (red) and SOX2 (grey) in sections of organoids co-cultured with H2B-GFP GSCs for 7 days. Green fluorescence marks GSCs and nuclei are counterstained with DAPI (blue). Higher magnification images in E–G and L–N show the invading front of GSCs migrating from the surface into the organoid wall (white arrows in D and K indicate the migrating front of GSCs). H2B-GFP GSCs positive for only SOX2 (blue arrowheads), only TUJ (white arrowheads) are indicated. Scale bars: A–D,H–K 50 µm; E–G,L–N 25 µm. (O) Quantification of the percentage of GFP+ cells in each of the six bins, over the total of GFP+ cells. Image on the left shows bin subdivision. (P) Quantification of the percentage of GFP+ cells expressing only SOX2, only TUJ or co-expressing SOX2 and TUJ, over the total of GFP+ cells in each bin. Data in O and P are presented as the mean±s.e.m. from three different GOCs obtained from three different experiments.

We asked whether GSCs in organoids would start differentiating or would maintain their stem-like phenotype. We cryosectioned the organoids after 7 days of co-culture with 1×10^4^ GSCs and performed immunostaining for the progenitor marker SOX2 and the neuronal marker TUJ (Fig. 4A–N). Our data show that the majority of the GFP+ tumor cells below the organoid surface express SOX2 (blue arrowheads in Fig. 4E–G,L–N indicate cells positive for only SOX2; for quantification see Fig. 4O,P). In contrast, the cells that migrated deeper into the organoid tissue, although a relatively minor fraction of all the engrafted cells (Fig. 4O, bins 4, 5 and 6), were mainly positive for the neuronal marker TUJ (white arrowheads in Fig. 4E–G,L–N mark cells positive for only TUJ; for quantification see Fig. 4P), indicating that cells tend to undergo differentiation as they invade the organoids.

Since organoids are kept in maturation conditions, co-cultured GSCs will be exposed to a medium that promotes differentiation. To examine the specific effect of organoid maturation media on GSC differentiation, we compare the fate of GSCs grown in 2D cultures in their usual neural stem cell growth media (plus EGF and FGF2) or exposed to the organoid maturation media (Fig. 5). We found that all GSCs express the neural stem cell marker SOX2 in both conditions (Fig. 5A,B). However, while only a few cells co-express SOX2 and TUJ in growth conditions (below 12%), 31%±2% of cells arranged in clusters co-expressed the two markers once exposed to the organoid maturation media (Fig. 5B,C), indicating that this medium is likely to promote differentiation while maintaining stemness.

**Fig.**
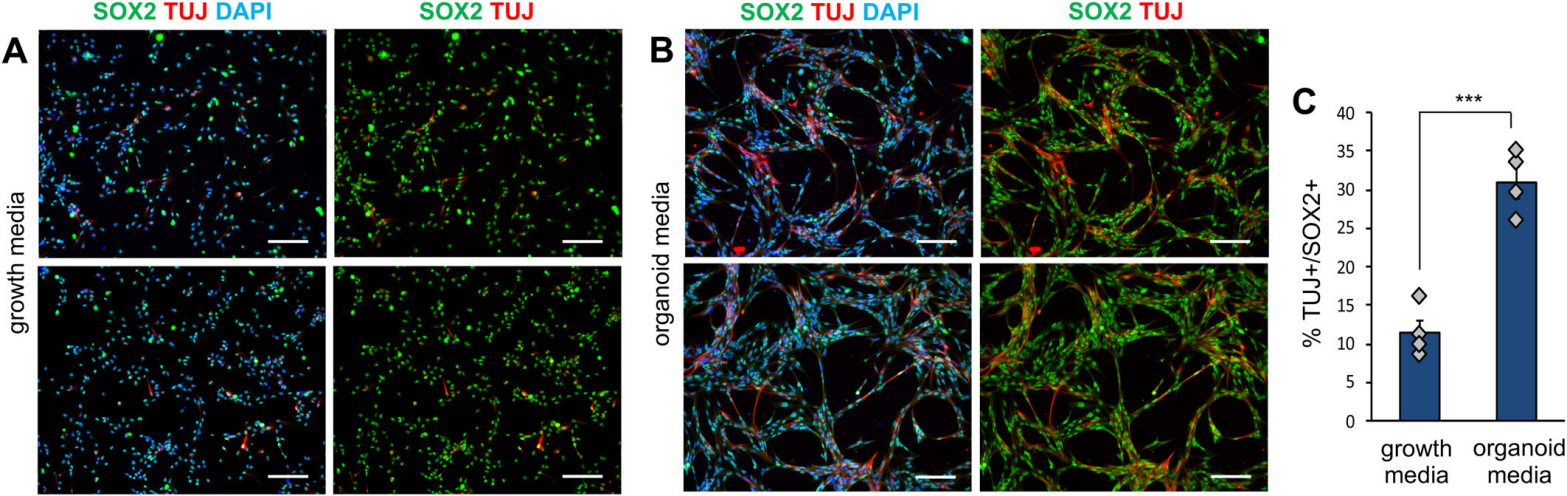
**5. Differentiation state of** **GSCs** **in organoid media.** (A,B) Immunostaining for TUJ and SOX2 in 2D culture of GSCs grown in either growth media (A) or organoid maturation media (B). Scale bars: 100 µm. (C) Quantification of the percentage of SOX2+ cells that co-express TUJ. *n*=4 *t*-test ***P<0,001.

In conclusion, the 3D spatial interaction of GSCs with organoid cells, together with the organoid media conditions generates a microenvironment that might allow GSCs to recapitulate developmental lineage progression programs and maintain the simultaneous presence of stem cells/progenitors and their more differentiated progeny.

## DISCUSSION

Modelling GBM in the lab is essential to advance our understanding of its biology and to develop new therapeutic strategies to treat such an aggressive type of tumor. Recently, researchers have started to develop methods to culture cancer cells in 3D, with the aim to better mimic the behavior of the *in vivo* tumor (reviewed in Azzarelli, 2020; Gomez et al., 2019; Silvia and Dai, 2020). This has been achieved by either introducing cancer cells or fragments of tumor biopsies directly in the semisolid matrix Matrigel (Hubert et al., 2016; Jacob et al., 2020), by genetically engineering brain organoids to activate oncogenes or delete tumor suppressors (Bian et al., 2018) or by co-culturing GSCs with 3D brain organoids (Linkous et al., 2019; Pine et al., 2020; Zhu et al., 2020). Here we describe a step-by-step guide to perform GSC-organoid co-culture, a model that has the advantage of maintaining the genetic complexity of the original tumor, while recreating an environment that is composed also of normal non transformed cells, as is found *in vivo*.

Optimal organoid generation is essential to provide the correct brain-like environment for glioblastoma cells. Human brain organoids can be generated from directed differentiation of human embryonic stem cells or induced pluripotent stem cells (Kadoshima et al., 2013; Lancaster et al., 2013) and can be regionalized to model specific brain regions (Cederquist et al., 2019; Dias and Guillemot, 2017; Qian et al., 2016; Renner et al., 2017). Here, we aimed to generate organoids with dorsal pallium identity, one of the common sites of GBM development, and used them as host ‘tissue’ for GSC growth and invasion. In the future, it would be interesting to test whether pre-patterning of organoids to other brain regions might influence GSC engraftment and behavior. In this study, we used three different iPSC lines and found that only two of them are capable of generating well-organized cerebral organoids, as assessed by morphological landmarks and immunochemical staining. It is not clear why the capacity to generate high quality organoids varies from one cell line to another, but genetic and epigenetic differences between the lines are likely to play a role (Ortmann et al., 2020; Volpato and Webber, 2020).

In parallel with the production of organoids, GSCs were expanded in 2D and fluorescently labelled so that they can be traced once integrated into the organoids. We followed the behavior of two different cell lines in our GOC system and found that GSCs with similar genetic alterations exhibit comparable behaviors upon organoid engraftment, in line with current reports (Linkous et al., 2019). In addition, our immunochemical analysis of GOCs shows that the majority of GSCs maintain their stem cell-like phenotype, while some GSCs start differentiating into the neuronal lineage. By allowing cells to differentiate as they would *in vivo*, our model can be readily used to study GSC heterogeneity and dynamic cell behavior (Bhaduri et al., 2020) and to test the selective trophism of viruses, such as ZIKV, for different normal and transformed cell types along the lineage (Zhu et al., 2020). The maintenance of a stem cell signature by GSCs in co-culture with organoids seems to be a peculiar feature of this model, as shown by recent single cell transcriptomic data comparing GSCs from different model types (Pine et al., 2020).

In the future, it will be important to establish how GSCs progress along their lineage at later time points in culture, and see whether the conditions can support GSC differentiation into glial cells. The addition of other cell types, such as immune cells (Abud et al., 2017; Brownjohn et al., 2018; Ormel et al., 2018), or the development of blood vessel-like structures (Daviaud et al., 2018; Mansour et al., 2018) within the organoids, might help to recreate the complex tumor microenvironment, and would likely make GSC lineage progression and response to treatment even more similar to the ones *in vivo*.

## MATERIALS AND METHODS

### hiPSC cultures

We used three cell lines: BobC and FSPS-13B cells from the BRC hiPSC core facility and the line IMR-90 from WiCell Research Institute (all lines were tested for contamination). BobC and FSPS-13B cells were cultured in Essential 8™ medium (Thermo Fisher Scientific, A1517001) on six-well plates pre-coated with vitronectin [STEMCELL Technologies, 7180, diluted 1:25 in PBS, for 1 h at room temperature (RT)]. The line IMR-90 was grown in StemFlex™ medium (Thermo Fisher Scientific, A3349401) on six-well plates pre-coated with Matrigel (Corning, 356231 0.5 mg per MW6, for 1 h at 37°C). Cells were dissociated using PBS-EDTA (0.5 mM) 5 min at RT every 5–7 days and re-plated 1:5 1:7. Cells were incubated at 37°C, 5% CO_2_.

### Generation of human cerebral organoids

Cerebral organoids were generated as described in (Lancaster et al., 2017), with minor modifications. We started from undifferentiated colonies of 80% confluent hiPSCs. Cells were detached using Accutase (Thermo Fisher Scientific, 00-4555-56) for 5 min at 37°C. Cells were counted in order to seed 9000 cells/well in ultra-low attachment 96-well plate (VWR, 7007). Cells were cultured in 100 µl/well of EB-media (Basal 1 plus supplement A, STEMCELL Technologies, 08570) mixed with microfibers (Ethicon suture vicryl, Aston Pharma, AP-ETW9567) and the ROCK inhibitor Y-27632 (Sigma-Aldrich, Y0503) 10 µM. No antibiotics were added at this stage. EB-media was added on day 2 and 4 to each well (90 µl/well). On day 5, EB-like aggregates were transferred to ultra-low attachment 24-well plate (VWR, 3473) (two aggregates/well) containing neural induction medium (Basal 1 plus Supplement B, STEMCELL Technologies, 8570).

On day 7, each organoid was embedded in Matrigel (Corning, 356234), as described in (Sutcliffe and Lancaster, 2017). Matrigel-organoid droplets were incubated for 30 min at 37°C to solidify the Matrigel. The droplets were then collected in 6 cm dishes (12 organoids/dish) and cultured in expansion media (Basal 2 plus Supplement C and D, STEMCELL Technologies, 08570).

On day 10, medium was changed to maturation medium (Basal 2 plus Supplement E, STEMCELL Technologies, 08571). Four days later, the Matrigel was removed and the organoids were cultured in 6 cm dishes in maturation medium on an orbital shaker for the rest of their time in culture. Medium was changed every 3–4 days and after day 30, Matrigel was dissolved in the medium (1%).

### Glioblastoma stem cell cultures

GSCs lines (GCGR-E27 and GCGR-E35, Glioma Cellular Genetic Resource, University of Edinburgh) were cultured in 10 cm dishes in growth media, as previously described (Pollard et al., 2009): D8437 media (Sigma-Aldrich), supplemented with Penicillin-Streptomycin (Thermo Fisher Scientific, 15140122), GlutaMAX™ (Thermo Fisher Scientific, 35050-061), 1% v/v N2 (Thermo Fisher Scientific, 17502-048), 2% v/v B27 (Thermo Fisher Scientific, 17504-044), 10 ng/ml bFGF (Peprotech, 100-18b), 10 ng/ml EGF (Peprotech, 315-09) and 1 µg/µl laminin (Sigma-Aldrich, L2020). Cells were maintained at 37°C and 5% CO_2_.

### Co-culture of GSCs and organoids

Cerebral organoids that have been in cultures for 42 days were transferred to ultra-low attachment 96-well plate (VWR, 7007; one organoid/well). Labelled GSCs (1×10^4^ or 5×10^4^ cells) were added to the wells and incubated overnight at 37°C. The day after, organoids and the engrafted GSCs were transferred back to a 6 cm dish in organoid maturation media. The GOC was then incubated at 37°C 5% CO_2_ on an orbital shaker for endpoint analysis at 1 or 7 days. Images and quantifications are from three organoids obtained from three different experiments.

### Organoid immunostaining

Organoids were fixed in PFA 4% for 30 min at RT and treated in 20% sucrose over-night at 4°C. Trypan Blue 1:100 was added for 10 min to facilitate organoid visualization during cryosectioning. Organoids were then embedded in OCT compound (VWR), and cryosectioned with a cryostat (Leica) at 12 µm thickness. Sections were permeabilized for 10 min at RT in 0.5% Triton X-100–PBS and blocked for 45 min at RT with 5% donkey serum (Sigma-Aldrich, D9663) in 0.01% Triton X-100–PBS. Sections were incubated over-night at 4°C with primary antibodies diluted in 2% donkey serum, 0.01% Triton X-100–PBS. Primary antibodies: goat anti-SOX2 (1:50, R&D, AF2018), goat anti-SOX17 (1:200, R&D, AF1924), mouse anti-NCAD (1:500 BD Biosciences, BD 610920), mouse anti-PAX6 (1:100 BD, 561462), mouse anti-TUJ (1:1000, Covance MMS-435P), rabbit anti-TBR1 (1:300, Abcam, ab31940). Fluorescently conjugated secondary antibodies Alexa Fluor (Thermo Fisher Scientific, 1:800) were added for 2 h at RT. For nuclear staining, sections were incubated with DAPI (Abcam) for 20 min at RT. Images were acquired with the confocal microscope SP5 (Leica) and quantified using ImageJ plugin cell counter.

### Immunocytochemistry of glioblastoma stem cells

GSCs were seeded in a 24-well plate (5×10^4^ cells/well) on a glass coverslip pre-coated with 1 µg/µl laminin (Sigma-Aldrich, L2020). GSCs were cultured in either growth media or in organoid maturation media for 2 days. GSCs were fixed in PFA 4% for 10 min and immunocytochemistry was performed as described in the ‘Organoid immunostaining’ section. Images were acquired with the fluorescence microscope Axiovert (Zeiss) and quantified after blind randomization using ImageJ plugin cell counter.

### Lentivirus production

Lentiviral particles were produced in HEK293T cells as described in (Azzarelli et al., 2018a). Briefly, HEK293T cells were transfected with third generation packaging vectors (vectors containing VSVG, gag-pol, rev and tat in a ratio 7:1:1.1) and with pBob-H2B-GFP vector (kind gift from Professor Rick Livesey), using Calcium Phosphate method (Promega ProFection^®^ kit, E1200). The morning after transfection, the medium was refreshed and then collected for viral production 36 h later. To concentrate the viral particles, the medium was mixed with the concentrator (Lenti-X™ Concentrator, Clontech, 63123) overnight at 4°C and then spun for 45 min at 2000× ***g*** at 4°C. After removing the supernatant, the pellet containing viral particles was resuspended in 200–300 µl of medium and stored in 20 µl aliquotes at −80°C. Viral titer was quantified using Titration kit (Lenti-X™ Titration kit, Clontech, 631235); the viral particles needed have been calculated using the formula: viral particle=[(total number of cells×MOI-multiplicity of infection)/titer)]×1000. The MOI used in our experiments is 10.

### Infection of glioblastoma stem cells

For lentiviral infection of GSCs, cells were plated in a 24-well plate (5×10^4^ cells/well). The day after plating, the medium was replenished with 300 µl of fresh medium containing the virus at MOI 10 with or without polybrene (8 µg/µl, Sigma-Aldrich, H9268). The day after infection, the medium was replaced with 500 µl of fresh media without virus.

### Transfection of glioblastoma stem cells

For transfection of GSCs, cells were plated in a 24-well plate (7×10^4^ cells/well). The day after plating, GSCs have been transfected using either Lipofectamine (Thermo Fisher Scientific, 11668027) or TransIT-LT1 (Mirusbio, MIR 2304), at 1:1 ratio of µg of DNA/µl of transfecting reagent.

## Supplementary Material

Supplementary information

## References

[BIO056416C1] Abud, E. M., Ramirez, R. N., Martinez, E. S., Healy, L. M., Nguyen, C. H. H., Newman, S. A., Yeromin, A. V., Scarfone, V. M., Marsh, S. E., Fimbres, C. et al. (2017). iPSC-derived human microglia-like cells to study neurological diseases. *Neuron* 94, 278-293.e9. 10.1016/j.neuron.2017.03.042 28426964 PMC5482419

[BIO056416C2] Aldape, K., Brindle, K. M., Chesler, L., Chopra, R., Gajjar, A., Gilbert, M. R., Gottardo, N., Gutmann, D. H., Hargrave, D., Holland, E. C. et al. (2019). Challenges to curing primary brain tumours. *Nat. Rev. Clin. Oncol.* 16, 509-520. 10.1038/s41571-019-0177-5 30733593 PMC6650350

[BIO056416C3] Azzarelli, R. (2020). Organoid models of glioblastoma to study brain tumor stem cells. *Front. Cell Dev. Biol.* 8, 220. 10.3389/fcell.2020.00220 32373607 PMC7176979

[BIO056416C4] Azzarelli, R., Rulands, S., Nestorowa, S., Davies, J., Campinoti, S., Gillotin, S., Bonfanti, P., Göttgens, B., Huch, M., Simons, B. et al. (2018a). Neurogenin3 phosphorylation controls reprogramming efficiency of pancreatic ductal organoids into endocrine cells. *Sci. Rep.* 8, 15374. 10.1038/s41598-018-33838-5 30337647 PMC6193982

[BIO056416C5] Azzarelli, R., Simons, B. D. and Philpott, A. (2018b). The developmental origin of brain tumours: a cellular and molecular framework. *Development* 145, dev162693. 10.1242/dev.162693 29759978 PMC6001369

[BIO056416C6] Bhaduri, A., di Lullo, E., Jung, D., Müller, S., Crouch, E. E., Espinosa, C. S., Ozawa, T., Alvarado, B., Spatazza, J., Cadwell, C. R. et al. (2020). Outer radial glia-like cancer stem cells contribute to heterogeneity of glioblastoma. *Cell Stem Cell* 26, 48-63.e6 10.1016/j.stem.2019.11.015 31901251 PMC7029801

[BIO056416C7] Bian, S., Repic, M., Guo, Z., Kavirayani, A., Burkard, T., Bagley, J. A., Krauditsch, C. and Knoblich, J. A. (2018). Genetically engineered cerebral organoids model brain tumor formation. *Nat. Methods* 15, 631-639. 10.1038/s41592-018-0070-7 30038414 PMC6071863

[BIO056416C8] Brownjohn, P. W., Smith, J., Solanki, R., Lohmann, E., Houlden, H., Hardy, J., Dietmann, S. and Livesey, F. J. (2018). Functional studies of missense TREM2 mutations in human stem cell-derived microglia. *Stem Cell Rep.* 10, 1294-1307. 10.1016/j.stemcr.2018.03.003 PMC5998752 29606617

[BIO056416C9] Cederquist, G. Y., Asciolla, J. J., Tchieu, J., Walsh, R. M., Cornacchia, D., Resh, M. D. and Studer, L. (2019). Specification of positional identity in forebrain organoids. *Nat. Biotechnol.* 37, 436-444. 10.1038/s41587-019-0085-3 30936566 PMC6447454

[BIO056416C10] Conti, L., Pollard, S. M., Gorba, T., Reitano, E., Toselli, M., Biella, G., Sun, Y., Sanzone, S., Ying, Q.-L., Cattaneo, E. et al. (2005). Niche-independent symmetrical self-renewal of a mammalian tissue stem cell. *PLoS Biol.* 3, e283. 10.1371/journal.pbio.0030283 16086633 PMC1184591

[BIO056416C11] Couturier, C. P., Ayyadhury, S., Le, P. U., Nadaf, J., Monlong, J., Riva, G., Allache, R., Baig, S., Yan, X., Bourgey, M. et al. (2020). Single-cell RNA-seq reveals that glioblastoma recapitulates a normal neurodevelopmental hierarchy. *Nat. Commun.* 11, 3406. 10.1038/s41467-020-17186-5 32641768 PMC7343844

[BIO056416C12] Daviaud, N., Friedel, R. H. and Zou, H. (2018). Vascularization and engraftment of transplanted human cerebral organoids in mouse cortex. *eNeuro* 5, ENEURO.0219-18.2018. 10.1523/ENEURO.0219-18.2018 PMC6243198 30460331

[BIO056416C13] Dias, C. and Guillemot, F. (2017). Revealing the inner workings of organoids. *EMBO J.* 36, 1299-1301. 10.15252/embj.201796860 28438893 PMC5430205

[BIO056416C14] Galli, R., Binda, E., Orfanelli, U., Cipelletti, B., Gritti, A., de Vitis, S., Fiocco, R., Foroni, C., Dimeco, F. and Vescovi, A. (2004). Isolation and characterization of tumorigenic, stem-like neural precursors from human glioblastoma. *Cancer Res.* 64, 7011-7021. 10.1158/0008-5472.CAN-04-1364 15466194

[BIO056416C15] Gomez, G. A., Oksdath, M., Brown, M. P. and Ebert, L. M. (2019). New approaches to model glioblastoma in vitro using brain organoids: implications for precision oncology. *Transl. Cancer Res.* 8, 1-6. 10.21037/tcr.2019.09.08 PMC8798484 35117142

[BIO056416C16] Goranci-Buzhala, G., Mariappan, A., Gabriel, E., Ramani, A., Ricci-Vitiani, L., Buccarelli, M., D'Alessandris, Q. G., Pallini, R. and Gopalakrishnan, J. (2020). Rapid and efficient invasion assay of glioblastoma in human brain organoids. *Cell Rep.* 31, 107738. 10.1016/j.celrep.2020.107738 32521263

[BIO056416C17] Hubert, C. G., Rivera, M., Spangler, L. C., Wu, Q., Mack, S. C., Prager, B. C., Couce, M., McLendon, R. E., Sloan, A. E. and Rich, J. N. (2016). A three-dimensional organoid culture system derived from human glioblastomas recapitulates the hypoxic gradients and cancer stem cell heterogeneity of tumors found in vivo. *Cancer Res.* 76, 2465-2477. 10.1158/0008-5472.CAN-15-2402 26896279 PMC4873351

[BIO056416C18] Jacob, F., Salinas, R. D., Zhang, D. Y, Nguyen, P. T. T., Schnoll, J. G., Zheng, S., Wong, H., Thokala, R., Sheikh, S., Saxena, D. et al. (2020). A patient-derived glioblastoma organoid model and biobank recapitulates inter- and intra-tumoral heterogeneity. *Cell* 180, 188-204.e22. 10.1016/j.cell.2019.11.03 31883794 10.1016/j.cell.2019.11.036 PMC7556703

[BIO056416C19] Kadoshima, T., Sakaguchi, H., Nakano, T., Soen, M., Ando, S., Eiraku, M. and Sasai, Y. (2013). Self-organization of axial polarity, inside-out layer pattern, and species-specific progenitor dynamics in human ES cell-derived neocortex. *Proc. Natl. Acad. Sci. USA* 110, 20284-20289. 10.1073/pnas.1315710110 24277810 PMC3864329

[BIO056416C20] Krieger, T. G., Tirier, S. M., Park, J., Jechow, K., Eisemann, T., Peterziel, H., Angel, P., Eils, R. and Conrad, C. (2020). Modeling glioblastoma invasion using human brain organoids and single-cell transcriptomics. *Neuro Oncol.* 22, 1138-1149. 10.1093/neuonc/noaa091 32297954 PMC7594554

[BIO056416C21] Lan, X., Jörg, D. J., Cavalli, F. M. G., Richards, L. M., Nguyen, L. V., Vanner, R. J., Guilhamon, P., Lee, L., Kushida, M. M., Pellacani, D. et al. (2017). Fate mapping of human glioblastoma reveals an invariant stem cell hierarchy. *Nature* 549, 227-232. 10.1038/nature23666 28854171 PMC5608080

[BIO056416C22] Lancaster, M. A., Renner, M., Martin, C.-A., Wenzel, D., Bicknell, L. S., Hurles, M. E., Homfray, T., Penninger, J. M., Jackson, A. P. and Knoblich, J. A. (2013). Cerebral organoids model human brain development and microcephaly. *Nature* 501, 373-379. 10.1038/nature12517 23995685 PMC3817409

[BIO056416C23] Lancaster, M. A., Corsini, N. S., Wolfinger, S., Gustafson, E. H., Phillips, A. W., Burkard, T. R., Otani, T., Livesey, F. J. and Knoblich, J. A. (2017). Guided self-organization and cortical plate formation in human brain organoids. *Nat. Biotechnol.* 35, 659-666. 10.1038/nbt.3906 28562594 PMC5824977

[BIO056416C24] Lee, J., Kotliarova, S., Kotliarov, Y., Li, A., Su, Q., Donin, N. M., Pastorino, S., Purow, B. W., Christopher, N., Zhang, W. et al. (2006). Tumor stem cells derived from glioblastomas cultured in bFGF and EGF more closely mirror the phenotype and genotype of primary tumors than do serum-cultured cell lines. *Cancer Cell* 9, 391-403. 10.1016/j.ccr.2006.03.030 16697959

[BIO056416C25] Linkous, A., Balamatsias, D., Snuderl, M., Edwards, L., Miyaguchi, K., Milner, T., Reich, B., Cohen-Gould, L., Storaska, A., Nakayama, Y. et al. (2019). Modeling patient-derived glioblastoma with cerebral organoids. *Cell Rep.* 26, 3203-3211.e5. 10.1016/j.celrep.2019.02.063 30893594 PMC6625753

[BIO056416C26] Louis, D. N., Perry, A., Reifenberger, G., von Deimling, A., Figarella-Branger, D., Cavenee, W. K., Ohgaki, H., Wiestler, O. D., Kleihues, P. and Ellison, D. W. (2016). The 2016 world health organization classification of tumors of the central nervous system: a summary. *Acta Neuropathol.* 131, 803-820. 10.1007/s00401-016-1545-1 27157931

[BIO056416C27] Lu, Q. R., Qian, L. and Zhou, X. (2019). Developmental origins and oncogenic pathways in malignant brain tumors. *Wiley Interdiscip. Rev. Dev. Biol.* 8, e342. 10.1002/wdev.342 30945456 PMC6565468

[BIO056416C28] Mansour, A. A., Gonçalves, J. T., Bloyd, C. W., Li, H., Fernandes, S., Quang, D., Johnston, S., Parylak, S. L., Jin, X. and Gage, F. H. (2018). An in vivo model of functional and vascularized human brain organoids. *Nat. Biotechnol.* 36, 432-441. 10.1038/nbt.4127 29658944 PMC6331203

[BIO056416C29] Neftel, C., Laffy, J., Filbin, M. G., Hara, T., Shore, M. E., Rahme, G. J., Richman, A. R., Silverbush, D., Shaw, M. L., Hebert, C. M. et al. (2019). An integrative model of cellular states, plasticity, and genetics for glioblastoma. *Cell* 178, 835-849.e21. 10.1016/j.cell.2019.06.024 31327527 PMC6703186

[BIO056416C30] Ogawa, J., Pao, G. M., Shokhirev, M. N. and Verma, I. M. (2018). Glioblastoma model using human cerebral organoids. *Cell Rep.* 23, 1220-1229. 10.1016/j.celrep.2018.03.105 29694897 PMC6892608

[BIO056416C31] Ormel, P. R., Vieira de Sá, R., van Bodegraven, E. J., Karst, H., Harschnitz, O., Sneeboer, M. A. M., Johansen, L. E., van Dijk, R. E., Scheefhals, N., Berdenis van Berlekom, A. et al. (2018). Microglia innately develop within cerebral organoids. *Nat. Commun.* 9, 4167. 10.1038/s41467-018-06684-2 30301888 PMC6177485

[BIO056416C32] Ortmann, D., Brown, S., Czechanski, A., Aydin, S., Muraro, D., Huang, Y., Tomaz, R. A., Osnato, A., Canu, G., Wesley, B. T. et al. (2020). Naive pluripotent stem cells exhibit phenotypic variability that is driven by genetic variation. *Cell Stem Cell* 27, 470-481.e6. 10.1016/j.stem.2020.07.019 32795399 PMC7487768

[BIO056416C33] Patel, A. P., Tirosh, I., Trombetta, J. J., Shalek, A. K., Gillespie, S. M., Wakimoto, H., Cahill, D. P., Nahed, B. V., Curry, W. T., Martuza, R. L. et al. (2014). Single-cell RNA-seq highlights intratumoral heterogeneity in primary glioblastoma. *Science* 344, 1396-1401. 10.1126/science.1254257 24925914 PMC4123637

[BIO056416C34] Pine, A. R., Cirigliano, S. M., Nicholson, J. G., Hu, Y., Linkous, A., Miyaguchi, K., Edwards, L., Singhania, R., Schwartz, T. H., Ramakrishna, R. et al. (2020). Tumor microenvironment is critical for the maintenance of cellular states found in primary glioblastomas. *Cancer Discov.* 10, 964-979. 10.1158/2159-8290.CD-20-0057 32253265 PMC10256258

[BIO056416C35] Pollard, S. M., Conti, L., Sun, Y., Goffredo, D. and Smith, A. (2006). Adherent neural stem (NS) cells from fetal and adult forebrain. *Cereb. Cortex* 16, i112-i120. 10.1093/cercor/bhj167 16766697

[BIO056416C36] Pollard, S. M., Yoshikawa, K., Clarke, I. D., Danovi, D., Stricker, S., Russell, R., Bayani, J., Head, R., Lee, M., Bernstein, M. et al. (2009). Glioma stem cell lines expanded in adherent culture have tumor-specific phenotypes and are suitable for chemical and genetic screens. *Cell Stem Cell* 4, 568-580. 10.1016/j.stem.2009.03.014 19497285

[BIO056416C37] Qian, X., Nguyen, H. N., Song, M. M., Hadiono, C., Ogden, S. C., Hammack, C., Yao, B., Hamersky, G. R., Jacob, F., Zhong, C. et al. (2016). Brain-region-specific organoids using mini-bioreactors for modeling ZIKV exposure. *Cell* 165, 1238-1254. 10.1016/j.cell.2016.04.032 27118425 PMC4900885

[BIO056416C38] Renner, M., Lancaster, M. A., Bian, S., Choi, H., Ku, T., Peer, A., Chung, K. and Knoblich, J. A. (2017). Self-organized developmental patterning and differentiation in cerebral organoids. *EMBO J.* 36, 1316-1329. 10.15252/embj.201694700 28283582 PMC5430225

[BIO056416C39] Robertson, F. L., Marqués-Torrejón, M.-A., Morrison, G. M. and Pollard, S. M. (2019). Experimental models and tools to tackle glioblastoma. *Dis. Models Mech.* 12, dmm040386. 10.1242/dmm.040386 PMC6765190 31519690

[BIO056416C40] Silvia, N. and Dai, G. (2020). Cerebral organoids as a model for glioblastoma multiforme. *Curr. Opin. Biomed. Eng.* 13, 152-159. 10.1016/j.cobme.2020.03.004 32355905 PMC7192544

[BIO056416C41] Singh, S. K., Hawkins, C., Clarke, I. D., Squire, J. A., Bayani, J., Hide, T., Henkelman, R. M., Cusimano, M. D. and Dirks, P. B. (2004). Identification of human brain tumour initiating cells. *Nature* 432, 396-401. 10.1038/nature03128 15549107

[BIO056416C42] Sutcliffe, M. and Lancaster, M. A. (2017). A simple method of generating 3D brain organoids using standard laboratory equipment. In *Organoids. Methods in Molecular Biology* (ed. K. Turksen), vol. 1576. Humana, New York, NY. 10.1007/7651_2017_2. 28361479

[BIO056416C43] Swartling, F. J., Čančer, M., Frantz, A., Weishaupt, H. and Persson, A. I. (2015). Deregulated proliferation and differentiation in brain tumors. *Cell Tissue Res.* 359, 225-254. 10.1007/s00441-014-2046-y 25416506 PMC4286433

[BIO056416C44] Tirosh, I., Venteicher, A. S., Hebert, C., Escalante, L. E., Patel, A. P., Yizhak, K., Fisher, J. M., Rodman, C., Mount, C., Filbin, M. G. et al. (2016). Single-cell RNA-seq supports a developmental hierarchy in human oligodendroglioma. *Nature* 539, 309-313. 10.1038/nature20123 27806376 PMC5465819

[BIO056416C45] Volpato, V. and Webber, C. (2020). Addressing variability in iPSC-derived models of human disease: guidelines to promote reproducibility. *Dis. Models Mech.* 13, dmm042317. 10.1242/dmm.042317 PMC6994963 31953356

[BIO056416C46] Zhu, Z., Mesci, P., Bernatchez, J. A., Gimple, R. C., Wang, X., Schafer, S. T., Wettersten, H. I., Beck, S., Clark, A. E., Wu, Q. et al. (2020). Zika virus targets glioblastoma stem cells through a SOX2-integrin αvβ5 axis. *Cell Stem Cell* 26, 187-204.e10 10.1016/j.stem.2019.11.016 31956038 PMC9628766

