# Supplementary material for: Three-dimensional model of glioblastoma by co-culturing tumor stem cells with human brain organoids
Source: Biol Open. 2021 Feb 22;10(2):bio056416. doi: 10.1242/bio.056416 (PMC7928227; doi:10.1242/bio.056416)
Supplement: Supplementary information [file biolopen-10-056416-s1.pdf]

Fig. S1 (related to Fig. 1)

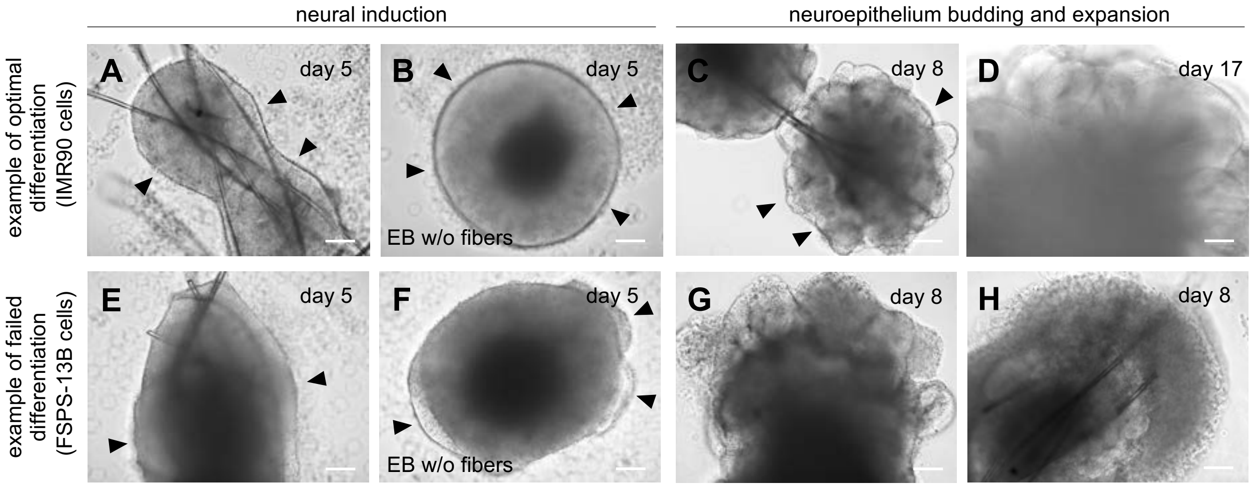

Fig S1. Morphological assessment of organoid quality. (A-D) Representative images showing the correct process of neural induction of IMR-90 cells with (A) or without (B) microfibres (bright neuroepithelium indicated by black arrowheads) and the correct formation and expansion of the neuroepithelial bulges (C-D, black arrowheads). (E-H) Images showing an example of incorrect neural induction of FSPS-13B cells with (E) or without (F) microfibres and incorrect neuroepithelial budding (G-H). Black arrowheads in E-F show irregular bulges within the forming neuroepithelium. EB: Embryoid Body. Scale bars: 100 µm

Fig. S2 (related to Fig. 2)

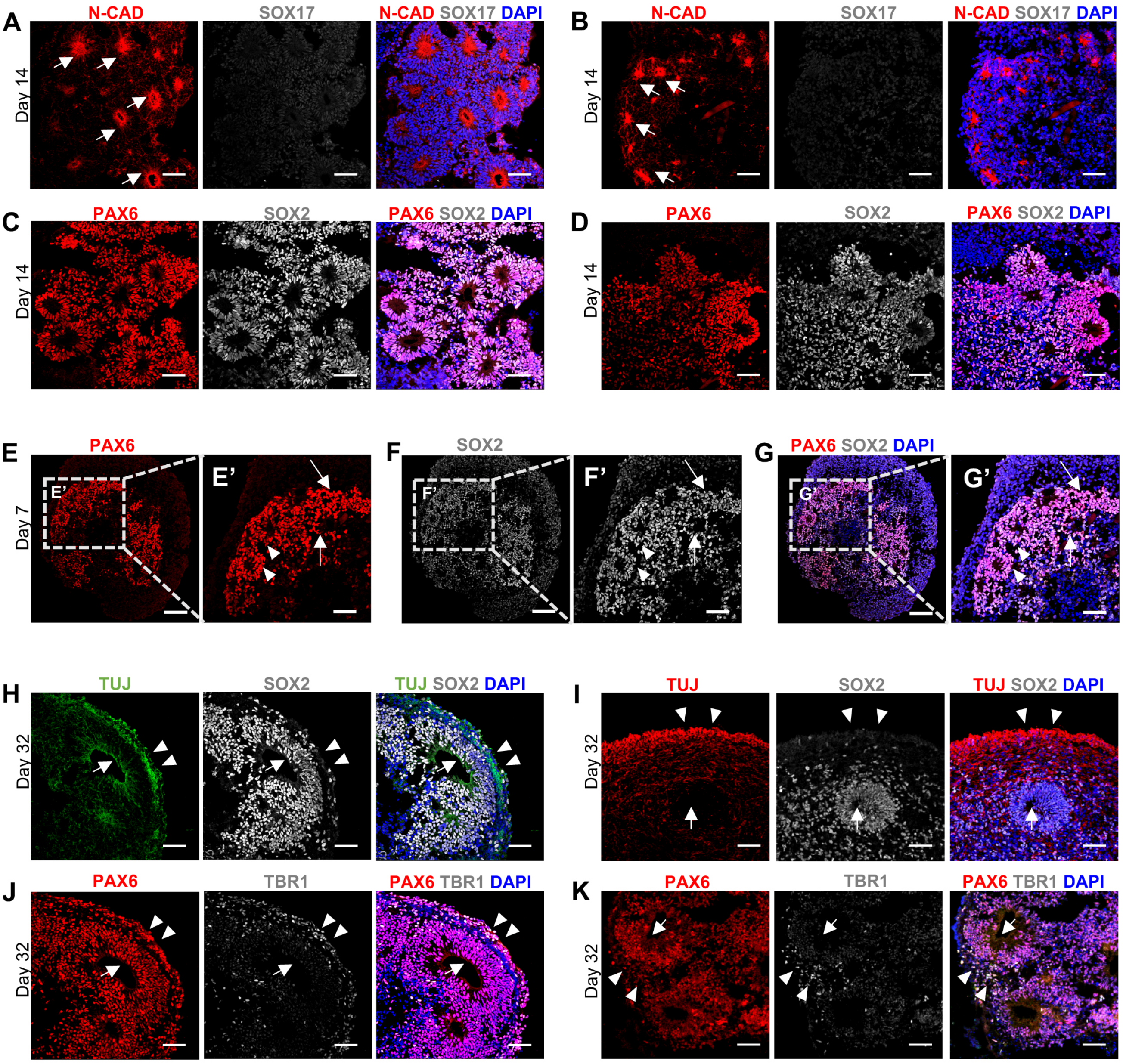

Fig S2. Molecular characterisation of cerebral organoids. (A-D) Immunostaining for N-CAD (red), SOX17 (grey) (A-B) and for PAX6 (red) and SOX2 (grey) (C-D) in organoid sections from 14 day old organoids. White arrows in A and B show N-CAD+ rosette-like structures. Scale bars: 50 µm. (E-G) Immunostaining for PAX6 (red) and SOX2 (grey) in aggregates that fail to generate organoids after 7 days in culture. (E'-G') Higher magnification of insets in E-G, showing rosette-like areas (white arrowheads) and spatially disorganised regions (white arrows). Scale bars: E-G 100 µm; E'-G' 50 µm. (H-K) Immunostaining for TUJ (green), SOX2 (grey) (H-I) and for PAX6 (red) and TBR1 (grey) (J-K) in 32 days old organoid sections. White arrows mark the ventricular areas, while white arrowheads indicate the outer surface. Scale bars: 50 µm

Biology Open • Supplementary information

Fig. S3 (related to Fig. 3)

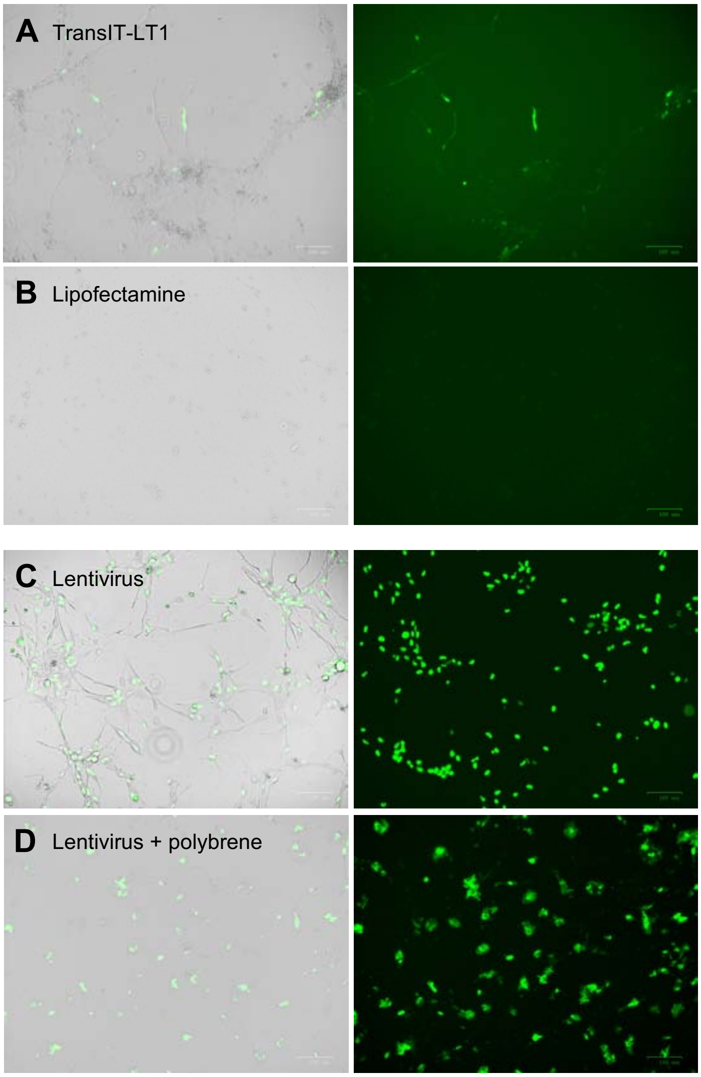

Fig. S3. Optimisation of glioblastoma stem cell fluorescent marking. (A-B) Transfection of GSCs: a GFP encoding plasmid has been transfected into GSCs via either TransIT-LT1 (A) or via Lipofectamine (B). (C-D) Viral transduction of GSCs: GSCs have been infected with lentiviruses carrying H2BGFP either without (C) or with polybrene (D). Images are phase contrast and green fluorescence. Scale bars: 100 µm

Biology Open • Supplementary information

Fig. S4 (related to Fig. 3)

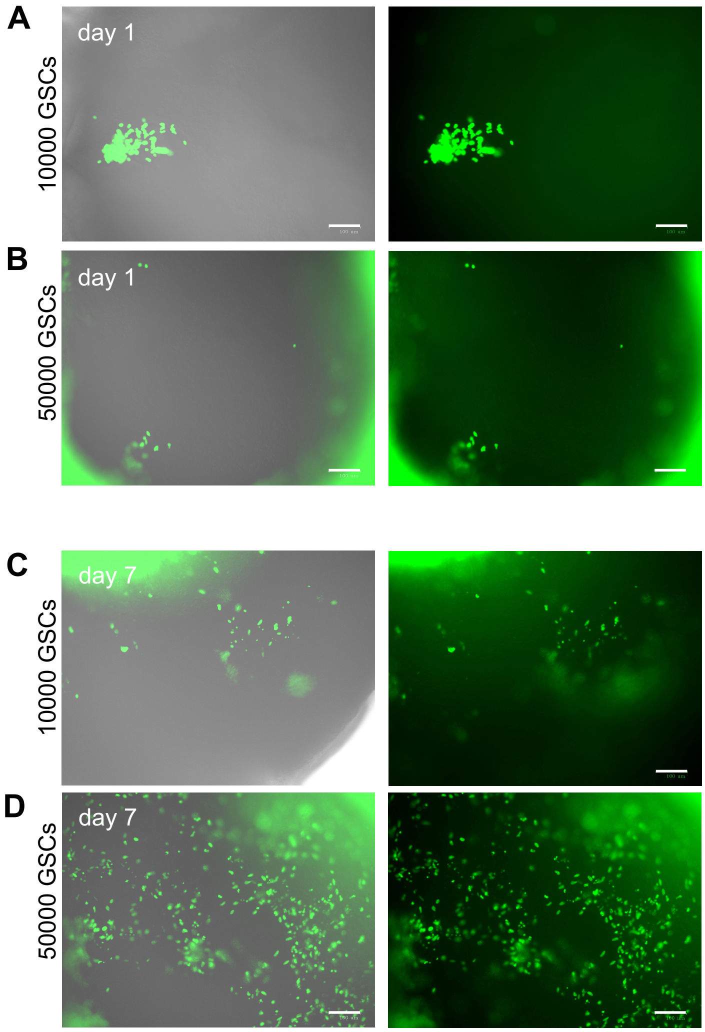

Fig. S4. Co-culture of glioblastoma stem cells (line GCGR-E35) and brain organoids. (A-B) Representative images of H2B-GFP GSCs (in green) invading brain organoids (in grey) at 1 day (A-B) and 7 days after co-culture (C-D). The number of GSCs used for the co-culture is indicated. n≥3 organoids. Scale bars: 100 µm.

Biology Open • Supplementary information
